# Social Support and Post-Injury Depressive and Anxiety Symptoms among College-Student Athletes

**DOI:** 10.3390/ijerph19116458

**Published:** 2022-05-26

**Authors:** Lindsay Sullivan, Kele Ding, Heather Tattersall, Sean Brown, Jingzhen Yang

**Affiliations:** 1 Discipline of Children’s Studies, School of Education, National University of Ireland, H91 TK33 Galway, Ireland; 2 Center for Injury Research and Policy, The Abigail Wexner Research Institute at Nationwide Children’s Hospital, Columbus, OH 43205, USA; heather.tattersall@gmail.com (H.T.); sean.brown2@nationwidechildrens.org (S.B.); ginger.yang@nationwidechildrens.org (J.Y.); 3 Department of Health Sciences, Kent State University, Kent, OH 44243, USA; kding@kent.edu; 4 Department of Pediatrics, College of Medicine, The Ohio State University, Columbus, OH 43205, USA

**Keywords:** anxiety, college-student athletes, depression, injury, social support

## Abstract

Social support can positively influence both physical and psychological recovery from sport-related injury. However, few studies have examined the influence of the quantity, quality, and timing of social support on athletes’ psychological health following injury. This study examined the effects of changes in social support on post-injury depressive and anxiety symptoms among college-student athletes. We conducted a prospective cohort study among Division I college-student athletes. Participants completed surveys at baseline and at multiple time points post-injury until return to play (RTP). A total of 597 injuries sustained by 389 student athletes (*n* = 400 (67.0%) males; *n* = 238 (39.9%) football players; *n* = 281 (47.1%) freshman) were included. The overall amount of social support increased from baseline to 1-week post-injury (*p* < 0.05) and then remained unchanged until RTP. The overall satisfaction with the support received increased from baseline to 1-week post-injury (*p* < 0.05) but decreased (*p* < 0.05) from 1-week post-injury to RTP. Increases in satisfaction with the support received were associated with decreases in post-injury depressive (β = −0.404), *p* < 0.0001) and anxiety symptoms (β = −0.406), *p* < 0.0001). Interventions involving social support may help hasten college-student athletes’ psychological recovery from injury.

## 1. Introduction

Injuries are common among college-student athletes [1], with an estimated 90% of student athletes sustaining at least one sport-related injury during their career [2]. Sport-related injuries, especially those that result in a significant time loss, can have a profound negative effect on one’s mental health, potentially triggering depression, anxiety, and other negative consequences, such as substance misuse and subclinical eating disorders [3,4,5]. Prior studies show that injured athletes report higher levels of depressive symptoms [6] and generalized anxiety [6,7] when compared to their non-injured counterparts. However, many college-student athletes often do not seek help for mental health problems due to a variety of reasons, including the stigma attached to mental health challenges and care seeking behaviors [8,9,10,11,12]. This underscores the critical need for strategies to help injured college-student athletes manage their anxiety and depressive symptoms post-injury.

Social support, defined as individuals on whom a person can rely on during periods of stress [13], is an important resource for athletes who are coping with an injury and may be protective against mental health challenges [14,15,16]. Social support could ‘‘buffer’’ the effect of stress on injured athletes and, thus, indirectly influence their emotional wellbeing. In other words, social support could first help mitigate the stressor–stress relationship such that an injured athlete with more social support post-injury tends to report a lower level of stress than those with limited support. Second, social support could help an injured athlete cope with the injury and improve motivation during rehabilitation. While there is some uncertainty regarding the timing and type of social support needed at various stages of the recovery process, previous research has supported the buffering effect of social support [17]. Prior studies have found that college-student athletes who perceive less social support are at greater risk for mental health problems, such as depression or anxiety, post-injury than their counterparts [16,18,19,20]. Yang et al. [21] found that higher levels of satisfaction with the social support received from team athletic trainers (ATs) during recovery from injury were associated with decreased depressive and anxiety symptoms among college-student athletes at return to play (RTP). Taken together, these findings suggest that high levels of social support, in combination with greater satisfaction with the social support received, may help improve the mental health and well-being of college-student athletes post-injury.

Despite the body of literature highlighting the role of social support in psychological recovery from injury [15,16,19,21,22], few studies have specifically examined changes in the quantity of social support (i.e., amount of social support from each source) and the satisfaction with the social support received from pre- to post-injury and if these changes influence depressive and anxiety symptoms overtime post-injury. The current study aimed to (1) describe changes in post-injury social support, including the amount of social support received from each source during recovery from injury and the degree of satisfaction with each source of support among injured Division I college-student athletes; and (2) assess the effects of these changes in social support on changes in depressive and anxiety symptoms throughout the course of recovery from baseline to RTP. We hypothesized that injured athletes with greater changes in the amount of social support received from each source and higher levels of satisfaction with the social support received from each source during the course of recovery would report lower depressive and anxiety symptoms than their counterparts. The findings of this study will help further our understanding of the role of social support on college-student athletes’ mental health outcomes post-injury. Such findings may also inform the development of strategies that help injured college-student athletes manage depressive and anxiety symptoms following injury.

## 2. Materials and Methods

### 2.1. Study Participants

Participants were male and female college-student athletes from two Big 10 Conference universities who were at least 18 years old and participated in 1 of 9 Division I NCAA-sponsored sports (i.e., men’s baseball, basketball, football, wrestling; women’s basketball, field hockey, soccer, softball, or volleyball) between 1 August 2007 and 31 December 2011. Written informed consent was obtained immediately prior to participation in the study. A total of 957 college-student athletes (90.2% participation rate) were prospectively enrolled in the study between the 2007–2008 and 2011–2012 academic years. Of these, 389 (40.7%) sustained at least 1 injury during the study period. This study received ethical approval from the Institutional Review Boards at the participating universities (IRB ID #: 200507776).

### 2.2. Study Procedures

After receiving approval from the head coach of each team, the research team introduced the study to potential athlete participants during a scheduled team meeting at the beginning of the season and invited them to participate in the study. Athletes who expressed interest in the study provided written consent and completed a preseason baseline survey that assessed depressive and anxiety symptoms, social support, and other study measures. We then prospectively followed enrolled athletes to identify injury incidence using the Sports Injury Monitoring System (SIMS), an ongoing injury surveillance system established for Big 10 Conference universities [23]. Injury was defined as a reportable injury that occurred in an organized sport activity, required medical attention, and restricted full sport participation for 1 or more days [24]. Upon the identification of an injury that met the study definition, we followed injured athletes at multiple intervals until RTP, with assessments conducted at ≤ 1-week post-injury, 1-, 3-, 6-, and 12-months post-injury, and at RTP. At each follow-up assessment, we collected data on injured athletes’ depressive and anxiety symptoms and social support, along with other study measures. Detailed descriptions on the study procedures are described elsewhere [19,21]. The present study, which is part of a larger study, assessed the effects of social support on depressive and anxiety symptoms measured at each time point post-injury.

### 2.3. Study Measures

We measured depressive symptoms at baseline (i.e., pre-injury) and post-injury using the 20-item Center for Epidemiological Studies Depression Scale (CESD) [25,26], a widely used depression screening tool with an internal consistency coefficient of 0.97 and test–retest reliability of 0.71 [25]. Respondents reported how often they experienced 20 symptoms of depression during the past week on a 4-point scale (0 = symptoms experienced less than once a week to 3 = symptoms experienced 5–7 days a week). A composite score was calculated by summing the responses to these 20 items (possible range = 0–80), with higher scores indicating more depressive symptoms [25,26].

We measured anxiety using the 40-item State–Trait Anxiety Inventory (STAI), which includes separate measures of state anxiety (20 items) and trait anxiety (20 items) [27,28]. Internal consistency coefficients for the scale have ranged from 0.86 to 0.95 and test–retest reliability coefficients have ranged from 0.65 to 0.75 over a 2-month interval [27]. The trait-anxiety subscale assesses how a person feels in general towards various situations that may influence anxiety levels on a 4-point Likert Scale (1 = almost never to 4 = almost always). The state-anxiety subscale asks participants to rate how they feel in the present moment about various situations that may influence anxiety levels, each rated on a 4-point Likert scale (1 = not at all to 4 = very much so). An overall trait-anxiety (possible range = 20–80) and state-anxiety (possible range = 20–80) score were calculated as the sum of responses for each item on the respective subscale, with higher scores indicative of a greater level of anxiety [27,28]. In this study, we assessed pre-injury anxiety status using the 20-item trait-anxiety scale, and post-injury anxiety status at each time point using the 20-item state-anxiety scale.

We defined social support as college-student athletes’ appraisal of the support from their social network and how satisfied they were with that support. We measured 2 dimensions of social support using the 6-item Social Support Questionnaire (SSQ6) at each time point: [29,30] (1) number of different sources that the injured athlete received help or support from and (2) degree of satisfaction with the social support received from each source. Each of the 6 items presented a scenario or situation from which injured student athletes may receive social support (e.g., “Whom could you really count on to be dependable when you need help?”, “Whom could you really count on to help you feel better when you are feeling generally down in the dumps?”). For each scenario, participants indicated (a) whether they received social support during their recovery and, if yes, from which of the following people the support was received: (1) family, (2) friend, (3) coach, (4) athletic trainer (AT), (5) teammate, (6) physician, (7) counselor, or (8) other; and (b) their degree of satisfaction with the social support received from each source using a 6-point Likert Scale (1 = very dissatisfied to 6 = very satisfied). In this study, we collapsed the coding for the different sources that provided social support into 3 sources for analysis: (1) family and friend support, (2) team support (i.e., team AT, coach, and teammates) and (3) other support (i.e., physician, counselor, other). ATs were included as part of team support because each sport team at the participating universities had a designated AT(s) for each athletic season. For each of the three sources, we used 2 variables to measure the social support received: (1) amount of social support, measured as average amount of social support received across the 6 scenarios; and (2) satisfaction with the social support received, measured as the average satisfaction score across the 6 scenarios. Internal reliabilities for the SSQ6 have been deemed excelled (α: 0.93–0.96) [31].

Data on college-student athletes’ demographic characteristics (e.g., university attended, sex, race, sport, history of injury), class year when injured, type of injury, and injury severity were also collected and included as covariates in the analysis.

### 2.4. Statistical Analysis

Data analysis was conducted using SAS version 9.4 (SAS Institute Inc. Cary, NC, USA). We used descriptive statistics to describe participant’s demographic characteristics as well as their baseline depressive and anxiety scores. Because depressive and anxiety scores were not normally distributed, we conducted nonparametric tests (i.e., Wilcoxon two-sample test and Kruskal–Wallis test) to compare mean differences in baseline depressive and anxiety scores across demographic characteristics. We conducted longitudinal analyses across the three time points (i.e., baseline, 1-week post-injury, and RTP) using linear models with repeated measures to assess changes in post-injury social support (i.e., from baseline to 1-week post-injury, and to RTP), including the amount of social support from each source and the degree of satisfaction with each source of social support.

We used linear mixed models (LMMs) to assess the effects of changes in social support, including the amount of and satisfaction with the social support received, on changes in post-injury depressive and anxiety scores during recovery. The LMMs were constructed with a 3-level structure: repeated measures of mental health symptoms at follow-ups (level 1) nested within each injury (level 2), which were further nested within each athlete (level 3). Since the depressive and anxiety symptom scores were highly skewed at each data point, a log transformation was conducted to ensure approximate normality. To assess the effects of each of the 3 sources of social support (i.e., family/friend, team, and other) on changes in post-injury depressive and anxiety scores, we performed separate analyses for each source and adjusted for baseline depression and anxiety symptom scores, social support before injury, and other covariates (e.g., race, college class standing at enrollment, sport, time loss due to injury). Due to the collinearity caused by high correlations between the 3 sources of social support, we first regressed the social support from each source against the other sources and then included and adjusted the residual obtained in our mixed models.

## 3. Results

### 3.1. Pre-Injury Depression and Anxiety Symptoms and Injury Characteristics

A total of 597 injuries sustained by 389 athletes were included, accounting for an average of 1.53 injuries per athlete. Injuries were most common among male athletes (*n* = 400, 67.0%), football players (*n* = 238, 39.9%), and freshman (*n* = 281, 47.1%) (Table 1). Most injuries were orthopedic injuries (*n* = 526, 88.1%), and of these, sprains (*n* = 293, 49.1%) were most common, followed by strains (*n* = 78, 13.1%) and fractures (*n* = 54, 9.0%). Approximately one-tenth of injuries (*n* = 71, 11.9%) were concussions. Most athletes (*n* = 237, 39.9%) returned to play within 1 week of injury, and 28.3% (*n* = 168) of athletes returned to play between 1-week and 1-month post-injury.

The average baseline scores for depression and anxiety symptoms were 10.8 (SD = 8.9) and 41.0 (SD = 10.2), respectively (Table 1). We found no statistically significant differences in baseline depression symptoms and anxiety scores by sex or injury severity (i.e., time loss due to injury). However, school year at enrollment and sport played were associated with anxiety scores, with freshmen and baseball players reporting lower average anxiety scores at baseline as compared to their counterparts. Non-white athletes, men’s basketball players, and women’s field hockey and volleyball players reported higher average depression symptom scores at baseline than their counterparts (Table 1).

### 3.2. Changes in Social Support throughout Recovery

While the average amount of social support from all three sources increased from baseline to 1-week post-injury (β = 0.16, 95%CI = 0.041, 0.287), no significant changes were observed from 1-week post-injury to RTP (Table 2). The average satisfaction score with the social support received significantly increased from baseline to 1-week post-injury (β = 0.24, 95%CI = 0.131, 0.353), followed by a significant decrease from 1-week post-injury to RTP (β = −0.15, 95%CI = −0.256, −0.035).

Injured athletes reported that family/friends were the primary source of social support throughout their recovery, with a higher average number of and a greater degree of satisfaction with the support received from family/friends, as compared to the other sources of support. Statistically significant changes were found in the amount of (β = 0.08, 95%CI = 0.011, 0.145) and satisfaction with the support received (β = 0.09, 95%CI = 0.044, 0.139) from family/friends from baseline to 1-week post-injury (Table 2). Injured athletes also reported significant increases in the amount of (β = 0.17, 95%CI = 0.082, 0.269) and satisfaction with the support received (β = 0.13, 95%CI = 0.071, 0.192) from others from baseline to 1-week post-injury. Satisfaction with the social support received from the team (β = 0.12, 95%CI = 0.025, 0.204) significantly increased from baseline to 1-week post-injury, while there were no statistically significant changes observed in the amount of social support received from the team during the same period (β = 0.11, 95%CI = −0.037, 0.263).

Injured athletes also reported decreased satisfaction with the support received from family/friends (β = −0.05, 95%CI = −0.098, −0.004), their team (β = −0.09, 95%CI = −0.181, −0.003), and others (β = −0.07, 95%CI = −0.129, −0.009) from 1-week post-injury to RTP. However, there were no statistically significant changes in the amount of social support received from each of the three sources of social support from 1-week post-injury to RTP (Table 2).

### 3.3. Effects of Changes in Social Support on Changes in Depression and Anxiety Symptoms Post-Injury

Greater satisfaction with the social support received from family/friends was associated with decreased post-injury depressive (β = −1.04, *p* = 0.01) and anxiety symptom scores (β = −0.90, *p* = 0.01), as was greater satisfaction with the social support received from one’s team ((β = 0.36, *p* = 0.01; β = −0.42, *p* = 0.01, respectively). However, we found no statistically significant effects of changes in the amount of social support from each source on changes in post-injury depressive symptoms or anxiety, after adjusting for potential covariates (Table 3).

## 4. Discussion

This study investigated the effects of changes in social support on changes in depressive and anxiety symptoms post-injury among injured college-student athletes. The main results showed that greater levels of satisfaction with the social support received were associated with decreased depression and anxiety symptoms post-injury. Results also revealed that the amount of and satisfaction with the social support received increased from baseline to 1-week post-injury. However, while the amount of social support from all sources remained unchanged from 1-week post-injury to RTP, the satisfaction with the support received decreased during this period. The findings of this study highlight the importance of social support on the mental health of injured athletes from injury onset to full recovery. Our findings may help inform the development of proactive strategies, including those involving social support, that aim to improve the mental health of injured college-student athletes during recovery.

An athlete’s level of social support is important to the recovery process [14,32,33]. Such support can act as a mediating force between stressor (the injury) and the resulting psychological state of stress, mediating this connection through the provision and restoration of sources that athletes may perceive to be lost due to their injury (e.g., incapacitation, social isolation, confidence, identity) [14,17,20,34]. Athletes who receive more social support may, thus, find it easier to maintain their pre-injury psychological state, facilitating a more successful injury recovery and return to sport [16].

We found that overall satisfaction with the social support received was associated with reduced post-injury depression and anxiety symptoms, as was the satisfaction with the support received from each of the three sources of social support. These findings, in line with others, [21,32,35] suggest that high-quality social support could help improve injured athletes’ mental health post-injury. Additional results from the present study showed no such relationships between the amount of social support and post-injury depression and anxiety symptoms. These findings suggest that the level of satisfaction with the social support received may have a greater influence on the mental health of college-student athletes post-injury than the amount of social support received. Thus, providing injured student athletes with the appropriate type and timing of social support throughout their recovery may increase their satisfaction with the support received, which, in turn, may improve injured student athletes’ mental health post-injury. Additionally, interventions involving social support should assess and identify injured college athletes’ support needs and then be tailored accordingly. Finally, injured college-student athletes should also be screened for mental health symptoms throughout recovery and be referred to appropriate mental health services as needed.

It is important to recognize that injured college-student athletes need social support from the time of injury until RTP, in order to hasten both the physical and psychological recovery from injury, with needs being heightened at certain points during the recovery process [32,36]. Our findings showed significant changes in the amount of and satisfaction with the social support received during recovery from injury, with the amount of social support received from family/friends and others and the degree of satisfaction with the support received from each of the three sources increasing from baseline to 1-week post-injury. However, such trends did not persist from 1-week post-injury to RTP. This is consistent with prior research, suggesting injured athletes are not supported beyond physical rehabilitation and that many injured athletes feel abandoned by their coaches and teammates during the recovery process [37,38]. Several factors may explain these observed results. First, injured athletes may seek social support immediately following injury. However, injured athletes may be less likely to seek social support during later stages in the recovery process, especially for mental health problems, due to the stigma attached to mental health problems or mental health care seeking. Second, although social support was provided to athletes throughout their recovery from injury, it is possible that the timing or type of support provided did not meet their needs over time post-injury. Third, injured athletes’ teammates or coaches may not fully understand the psychological aspects of sports injury and the rehabilitation process, which may result in them unintentionally providing less or lower-quality support to the injured athlete after the acute injury period. Future qualitative studies are needed to explore reasons for the reduced amount of social support and decreased levels of satisfaction with the social support received from 1-week post-injury to RTP.

Consistent with prior studies [19,39], we found that family/friends were the main source of social support throughout recovery. This is not surprising, since athletes rely on their family/friends for support, both before and after injury. These results highlight how family/friends are a reliable and consistent source of social support. Teammates and coaches are another important source of support for injured athletes during recovery from injury, especially for injured college-student athletes who are living away from home. Social support from one’s teammates and coaches may be particularly important during the COVID-19 pandemic as injured athletes may not be able to see their family/friends in-person during the athletic season or due to the mental health challenges associated with the pandemic itself [40,41]. It is, therefore, crucial that one’s teammates and coaches provide high levels of social support to injured athletes throughout their recovery. Our findings call for more education for college-student athletes and coaches on the role of social support in psychological recovery from injury, as well as for behavioral training on how to provide high-quality emotional/instrumental support to injured athletes. Injured college-student athletes may also benefit from peer support groups with other injured college-student athletes. Peer support groups could provide injured college-student athletes with the opportunity to give and receive support from others in similar situations.

This study had several limitations that warrant attention. First, our findings are limited by the collection of data from a sample of college-student athletes from two Big 10 universities. Therefore, our findings may not be generalizable to other universities, divisions, or leagues. Second, our sample was limited by the unbalanced distribution of participants by sex, sport played, and class year when injured. Third, we measured perceived social support as opposed to the actual social support received throughout recovery. While perceived support has a greater influence on the outcome, our results should be interpreted in this light. Fourth, it is important to acknowledge that some unmeasured factors, such as mental healthcare seeking or sport modality, may have influenced the observed relationships between social support and post-injury depressive and anxiety symptoms; thus, the findings of this study should be interpreted with caution. Fifth, depression and anxiety symptoms were based on self-reported data and may have been subject to social desirability bias. Finally, the data presented in this study were collected 10 years ago and may be outdated. It is important to note, however, that to the best of our knowledge, no similar studies have been conducted since the collection of our study data. Despite these limitations, this study provides important insight into the influence of social support on the mental health of Division I college-student athletes following injury.

## 5. Conclusions

Our findings indicate that injured athletes reported an increase in the amount of social support and satisfaction with the social support received from baseline to 1-week post-injury. However, such a trend did not persist from 1-week post-injury to RTP. Our results also indicated that greater satisfaction with the social support received was associated with decreased post-injury depressive and anxiety symptoms. These findings suggest that interventions that involve social support may help injured college-student athletes manage depressive and anxiety symptoms post-injury. Future interventions could provide behavioral training to student athletes and coaches to equip them with the requisite skills to provide emotional/instrumental and informational support to injured athletes throughout the course of recovery.

## Figures and Tables

**Table 1:** Baseline characteristics and associated depression and anxiety symptoms.

	Overall	Depression Symptoms		Anxiety	
	% (*n*)	Mean (SD)	*p*-Value	Mean (SD)	*p*-Value
**Total**	597	10.8 (8.9)		41.0 (10.2)	
**Sex**			0.2809 ^b^		0.3661 ^b^
Male	67.0 (400)	10.7 (9.2)		41.3 (10.2)	
Female	33.0 (197)	11.0 (8.4)		40.4 (10.3)	
**Race ^a^**			0.0005 ^b^		0.6455 ^b^
White	74.5 (445)	10.0 (8.3)		40.8 (10.3)	
Non-white	25.3 (151)	13.3 (8.3) ^b^		41.4 (10.1)	
**School Year at Enrollment**			0.3696 ^c^		<0.0001 ^c^
Freshman	47.1 (281)	11.0 (8.5)		37.9 (9.8)	
Sophomore	20.6 (123)	10.5 (9.2)		42.0 (10.5)	
Junior	19.9 (119)	11.2 (9.3)		45.0 (9.2)	
Senior	12.4 (74)	9.9 (9.6)		44.4 (9.6)	
**Sport**			0.0008 ^c^		<0.0001 ^c^
Baseball (M)	4.9 (29)	6.3 (4.3)		33.7 (7.5)	
Basketball (M)	2.5 (15)	16.4 (11.7)		41.5 (10.1)	
Football (M)	39.9 (238)	11.0 (9.2)		41.1 (10.0)	
Wrestling (M)	19.9 (119)	10.5 (9.3)		43.4 (10.3)	
Basketball (W)	8.7 (52)	9.0 (7.8)		40.3 (11.2)	
Field Hockey (W)	5.7 (34)	13.5 (9.1)		40.7 (9.7)	
Soccer (W)	6.9 (41)	8.6 (6.8)		37.5 (9.1)	
Softball (W)	6.4 (38)	10.9 (7.6)		39.6 (10.8)	
Volleyball (W)	5.2 (31)	15.2 (9.4)		45.3 (9.3)	
**Time-Loss Due to** **Injury ^a^**			0.5091 ^c^		0.9146 ^c^
Time-loss ≤ 1 week	39.9 (237)	10.5 (8.8)		40.6 (10.1)	
1 week < Time-loss ≤ 1 month	28.3 (168)	10.5 (8.8)		41.6 (10.4)	
1 month < Time-loss ≤ 3 months	14.8 (88)	11.7 (8.8)		40.9 (9.5)	
3 months < Time-loss ≤ 6 months	10.6 (63)	12.2 (10.2)		41.2(11.1)	
Time-loss > 6 months	6.4 (38)	9.0 (6.8)		40.3 (11.0)	

Note: ^a^ A total less than 597 is due to missing; ^b^ *p* value was based on Wilcoxon two-sample test; ^c^ *p* value was based on Kruskal–Wallis test.

**Table 2:** Changes in social support from baseline, through 1-week post-injury, to return to play.

Changes in Social Support Post-Injury					
	Baseline	1-Week Post-Injury	Change from Baseline to 1-Week Post-Injury	Return to Play (RTP)	Change from 1-Week Post-Injury to RTP
	β (SE)	β (SE)	Difference (95%CI) ^2^	β (SE)	Difference (95%CI) ^2^
**Amount of social support Received**					
Overall	3.29 (0.072)	3.46 (0.072)	**0.16 (0.041, 0.287)**	3.41 (0.071)	−0.05 (−0.168, 0.080)
Family and Friends	5.16 (0.032)	5.24 (0.032)	**0.08 (0.011, 0.145)**	5.24 (0.032)	−0.01 (−0.067, 0.060)
Team ^1^	3.44 (0.070)	3.55 (0.070)	0.11(−0.037, 0.263)	3.49 (0.070)	−0.07 (−0.217, 0.080)
Others ^1^	1.31 (0.055)	1.49 (0.055)	**0.17 (0.082, 0.269)**	1.47 (0.054)	−0.02 (−0.119, 0.007)
**Satisfaction with Social Support Received**					
Overall	3.71 (0.059)	3.95 (0.059)	**0.24 (0.131, 0.353)**	3.81 (0.059)	**−0.15 (−0.256, −0.035)**
Family and Friends	5.24 (0.026)	5.33 (0.026)	**0.09 (0.044, 0.139)**	5.28 (0.026)	**−0.05 (−0.098, −0.004)**
Team ^1^	4.19 (0.045)	4.30 (0.045)	**0.12 (0.025, 0.204)**	4.22 (0.045)	**−0.09 (−0.181, −0.003)**
Others ^1^	1.71 (0.033)	1.84 (0.033)	**0.13 (0.071, 0.192)**	1.77 (0.033)	**−0.07 (−0.129, −0.009)**

^1^ Team included coach(s), athletic trainer(s), and teammates; others included physician, consoler, and others. ^2^ Differences in social support were assessed between baseline vs. 1-week post-injury and 1-week post-injury vs. return to play using longitudinal analysis with repeated measures.

**Table 3:** Effects of changes in social support on changes in depression and anxiety symptoms post-injury.

	Depression Symptoms			Anxiety		
	β (B^b^)	SD (SE)	P ^2^	β (B^b^)	SD (SE)	P ^2^
**Amount of social support Received**						
Overall	0.12	0.22	0.58	0.20	0.26	0.44
Family and Friends	−0.37	0.26	0.16	−0.13	0.33	0.69
Team ^1^	0.15	0.08	0.07	0.08	0.10	0.39
Others ^1^	−0.03	0.16	0.87	0.17	0.19	0.37
**Satisfaction with the Social Support Received**						
Overall	−4.04	0.48	**<0.0001**	−4.96	0.60	**<0.0001**
Family and Friends	−1.04	0.29	**0.01**	−0.90	0.36	**0.01**
Team ^1^	−0.36	0.11	**0.01**	−0.42	0.13	**0.01**
Others ^1^	−0.45	0.22	**0.04**	−0.22	0.27	0.43

^1^ Team included coach(s), athletic trainer(s), and teammates; others included physician, consoler, and others. ^2^ Linear mixed models were used to assess the effects of changes in social support from baseline to return to play on the changes in post-injury depressive and anxiety symptom scores during the course of recovery, adjusting for baseline depression and anxiety symptom score, baseline social support, and other covariates (e.g., race, college class standing at enrollment, sport, time loss due to injury).

## Data Availability

The data presented in this study are available on reasonable request from Jingzhen Yang (ginger.yang@nationwidechildrens.org). The data are not publicly available.
